# Using Excimer Laser for Manufacturing Stimuli Responsive Membranes

**DOI:** 10.3390/membranes13040398

**Published:** 2023-03-31

**Authors:** Erol Sancaktar

**Affiliations:** School of Polymer Science and Polymer Engineering, The University of Akron, Akron, OH 44325, USA; erol@uakron.edu

**Keywords:** stimuli responsive membrane, polymer membrane thickness effect, KrF excimer laser, pulsed laser polymerization and grafting, poly(N-isopropylacrylamide), permeability, controlled solute transfer, laser ablative pore creation

## Abstract

A 248 nm KrF excimer laser can be used to manufacture temperature and pH-responsive polymer-based membranes for controlled transport applications. This is done by a two-step approach. In the first step, well-defined/shaped and orderly pores are created on commercially available polymer films by ablation by using an excimer laser. The same laser is used subsequently for energetic grafting and polymerization of a responsive hydrogel polymer inside the pores fabricated during the first step. Thus, these smart membranes allow controllable solute transport. In this paper, determination of appropriate laser parameters and grafting solution characteristics are illustrated to obtain the desired membrane performance. Fabrication of membranes with 600 nm to 25 μm pore sizes by using the laser through different metal mesh templates is discussed first. Laser fluence and the number of pulses need to be optimized to obtain the desired pore size. Mesh size and film thickness primarily control the pore sizes. Typically, pore size increases with increasing fluence and the number of pulses. Larger pores can be created by using higher fluence at a given laser energy. The vertical cross-section of the pores turns out to be inherently tapered due to the ablative action of the laser beam. The pores created by laser ablation can be grafted with PNIPAM hydrogel by using the same laser to perform a bottom-up grafting-from type pulsed laser polymerization (PLP) in order to achieve the desired transport function controlled by temperature. For this purpose, a set of laser frequencies and pulse numbers need to be determined to obtain the desired hydrogel grafting density and the extent of cross-linking, which ultimately provide controlled transport by smart gating. In other words, on-demand switchable solute release rates can be achieved by controlling the cross-linking level of the microporous PNIPAM network. The PLP process is extremely fast (few seconds) and provides higher water permeability above the lower critical solution temperature (LCST) of the hydrogel. Experiments have shown high mechanical integrity for these pore-filled membranes, which can sustain pressures up to 0.31 MPa. The monomer (NIPAM) and cross-linker (mBAAm) concentrations in the grafting solution need to be optimized in order to control the network growth inside the support membrane pores. The cross-linker concentration typically has a stronger effect on the temperature responsiveness. The pulsed laser polymerization process described can be extended to different unsaturated monomers, which can be polymerized by the free radical process. For example, poly(acrylic acid) can be the grafted to provide pH responsiveness to membranes. As for the effects of thickness, a decreasing trend is observed in the permeability coefficient with increasing thickness. Furthermore, the film thickness has little or no effect on PLP kinetics. The experimental results have shown that membranes manufactured by excimer laser are excellent choices for applications where flow uniformity is the prime requirement, as they possess uniform pore sizes and distribution.

## 1. Introduction

Several different techniques have been used for membrane fabrication. These include the following. (1) Phase inversion: This process can be in the form of precipitation from the vapor phase or controlled evaporation, thermal precipitation, and immersion precipitation [1,2,3,4]. These processes basically involve solvent exchange and precipitation of a polymer followed by the evaporation of a nonsolvent and pore formation [2]. The phase inversion method typically results in nonuniform pore size, thus increasing tortuosity of the solute transport path, making it difficult to predict the transport rate [5]. (2) Stretching: This process involves hot stretching of an extruded semicrystalline polymer film, which acquires pores by lamellar separation [6,7,8]. It typically results in a nonuniform distribution of pore areas due to the nature of semicrystalline structures, thus leading to nonuniform solute transport over the membrane surface. (3) Interfacial polymerization: This method involves polymerization of a reactive monomer at the interface of two immiscible solvents [9] and typically results in nonuniform pore size, thus making it difficult to predict the transport rate. (4) Electrospinning: This involves a nonwoven fabrication technique that uses a high-voltage power source along with a syringe pump, a ground collector, and a spinneret to spin a polymer solution into nonwoven fabric form. Membranes with pore sizes varying from 10 nanometers to several micrometers can be produced in this manner. This method typically results in nonuniform pore size, thus making it difficult to predict the transport rate, as well as nonuniform distribution of pore areas leading to nonuniform solute transport over the membrane surface. (5) Track etching: With this method, a polymer film is irradiated by using high energy from fission of heavy nuclei or the use of ion beams for acceleration to create tracks on the film [10,11]. Pores are subsequently created and widened by using chemicals. With this process, the possibility of pore overlapping may result in a nonuniform flux through the surface of the membrane [12]. It is also noted that track etching, as well as phase inversion, interfacial polymerization, and electrospinning, all involve the use of environmentally undesirable solvents. (6) Laser lithography: Li and Sancaktar [13,14] used 248 nm KrF excimer laser irradiation on different thickness PET films (72 μm and 124 μm), over which different opening-sized (30 μm and 43 μm) stainless steel mesh masks were placed to manufacture differently sized perforated patterns and provide an effective and controllable perforation technology. For this purpose, a different laser energy fluence and number of laser pulses were used, and the morphology of irradiated samples was observed by optical microscopy and atomic forced microscopy to study topography and microstructures after laser ablation. The findings were correlated with the orientation, strength and mechanical properties of the polymer film. Their results showed that increasing the number of pulses increased the perforation size as well as the percentage of the membrane’s perforated area. The perforation size increased sharply initially, possibly due to the related decrease in PET crystallinity. The perforated area reached a plateau at a high number of pulses. The pulse number threshold for perforation decreased with increasing fluence. Catiker et al. used the ablative procedures reported by Li and Sancaktar [13,14] to modify surfaces of poly(3-hydroxybutyrate) (PHB), poly(lactic acid) (PLA), poly(methyl methacrylate) (PMMA), and polyurethane (PU)/poly(dimethylsiloxane) (PDMS) films by using the same 248 nm KrF excimer laser to produce cavities and orderly perforated holes on the films for tissue engineering by examining cell–polymer film interfacial interactions based on the proliferation of human fibroblast cells cultured on the laser-modified membranes in comparison to unexposed membranes and tissue culture dishes as controls [15].

**Figure 1:**
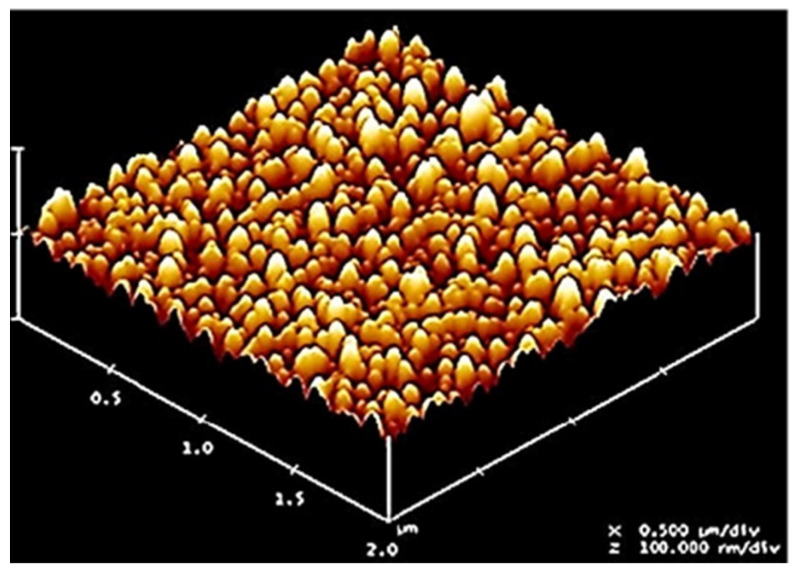
Mask-image-like high-density nanodots produced on the surfaces of silicon substrates by excimer laser ablation at 130 mJ/cm^2^ fluence on the surfaces of ~100 nm-thick SIS + PS12k and SIS + PS15k films as the thin block copolymer masks were completely removed after 100 pulses.

The size of the pores generated by excimer laser ablation, as described above [13,14,15] for flux in the direction perpendicular to the planar surface of the polymer film, can be reduced down to the nanoscale for flux in directions transverse to the film (i.e., in the film plane), again by using the excimer laser in conjunction with an innovative self-assembly technique. Polymer films processed in this manner can be stacked on top of each other and can even be incorporated with responsive hydrogels by pulsed laser polymerization (PLP) within the channels created on their surfaces (preferably prior to stacking) to be used as membranes with flux in their planar direction. Ahn and Sancaktar [16,17,18,19] illustrated the manufacture of densely packed polystyrene (PS) and silicon nanodots by using a top-down/bottom-up hybrid method by employing excimer laser irradiation on perpendicular cylinder-containing block copolymer films. For this purpose, the block copolymer self-assembly process was employed first [20,21,22,23], providing perpendicular PS nanocylinders in the polyisoprene (PI) matrix by control of segregation forces between the PS and PI block domains. By the preferential ablation of more ultraviolet (UV)-sensitive block component (PI), along with nonselective removal of all block components which reduced the overall sample thickness, the upright (perpendicular) PS nanopatterns in block copolymer masks were transferred to a silicon substrate, and the process labelled as a “one-step process”. We note that these two processes (ablation of PI and creation of nanodots in silicone) simultaneously occurred during excimer laser irradiation at appropriate laser intensity. Their numerical analysis, based on photothermal excimer laser ablation of nanostructured block copolymer masks, revealed that a sufficiently low laser intensity was appropriate for suppressing the surface melt flows of block components, which may disrupt the order of nanopatterns in the block copolymer masks, as long as the intensity was high enough to induce a matrix-assisted photothermal excimer laser ablation in less UV-sensitive block component (PS) [16,17]. Ahn and Sancaktar showed that by excimer laser ablation at 130 mJ/cm^2^ fluence on the surfaces of ~100 nm-thick SIS + PS12k and SIS + PS15k films, the thin block copolymer masks were completely removed after 100 pulses, and consequently mask-image-like high-density nanodots with (down to) ~5 nm- and ~3 nm-height were produced on the surfaces of silicon substrates, respectively, without considerable surface melt flows of block domains (see Figure 1) [16,17]. 

Thus, work by Ahn and Sancaktar showed that during the excimer laser irradiation on the regularly nanopatterned block copolymer masks, both block components were simultaneously removed even at the laser intensity lower than the ablation threshold of the less UV-sensitive block component. Obviously, this nanodot fabrication technique, which utilizes one-step excimer laser irradiation on block copolymer masks is simple and fast and has the potential to provide new opportunities for the fabrication of low-cost and high-throughput nanodots on the surfaces of functional organic/inorganic materials for membrane applications [16,17]. 

**Figure 2:**
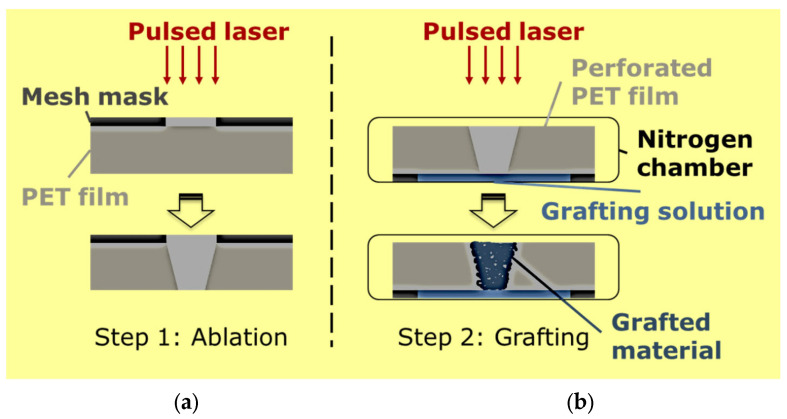
Manufacturing stimuli-responsive polymer-based membranes for controlled transport applications by using a two-step approach. In the first step, well-defined/shaped and orderly pores are created on commercially available polymer films by ablation by using an excimer laser (**a**). The same laser is used subsequently for energetic grafting and polymerization of a responsive hydrogel polymer inside the pores fabricated during the first step (**b**).

Sancaktar and coworkers recently introduced the use of the excimer laser for fabrication of stimuli-responsive membranes with polymer carrier films and hydrogel gating accomplished in the polymer pores fabricated by laser ablation [24,25,26,27,28,29]. The hydrogel is processed by using PLP, and the same excimer laser instrument can be used for both of these fabrication steps (Figure 2). This provides a highly efficient, fast, and accurate process that is superior to the existing membrane manufacture methods, including phase inversion, stretching, electrospinning, and interfacial polymerization.

## 2. Excimer Lasers

The precise ability to control the laser beam led to its extensive use in industrial and medical fields [30]. Various beam wavelengths can be obtained by using different gas mixtures in an excimer laser. This enables its use in modifying different polymer parts that have different ablation thresholds as well as in PLP processes.

Absorption of laser energy above a certain fluence (F), energy per unit area can lead to the removal of material from a polymer substrate by ablation [31,32]. The number of pulses (P), laser frequency (Hz), the absorption coefficient of the material, and experimental factors can all affect the process of ablation. Beer’s law (Equation (1)) defines the ablation depth, d [13,14,15,33,34,35],

*d* = (*1*⁄*α*) *ln*(*f*/*f_fth_*), (1)

where, *d* is etched depth (cm), α is absorption coefficient (cm^−1^), *f* is incident fluence (J/cm^−2^), and *f_th_* is the ablation threshold. 

Above the ablation threshold, the broken molecules are ejected from the irradiated region with the kinetic energy they acquire [36]. Ablation processes for ABS, PA6, PMMA, PI, PC, PS, PVA, PET, PP, etc., have been reported in the literature [33,34,37,38,39,40].

## 3. Pulsed Laser Polymerization (PLP)

PLP is frequently used to determine the propagation of rate coefficient *k_p_* [41]. Laser pulses create free radicals, which decrease in concentration due to termination during polymerization [42,43]. Polymer chain length ($L_{1}$) growth between two successive pulses is given by

(2) $$L_{1}=k_{p}*\left[M\right]*t_{0},\;\;\;\;\;\;\;\;\;$$

where $k_{p}$ is rate propagation coefficient, $t_{0}$ is the dark time between two successive pulses, and $\left[M\right]$ is monomer concentration. The polymer chain continues to grow during the dark time to a length which is multiple of $L_{0}$ if its free radical does not terminate. Thus, after ‘*n*’ pulses, the chain length ($L_{n}$) is given by

(3) $$L_{n}=n*k_{p}*\left[M_{n}\right]*t_{0}.$$

PLP has been used for polymers like acrylamide, acrylic acid, methyl methacrylate, and butyl acrylate [41,42,43,44,45,46,47,48,49,50,51,52,53]. 

## 4. Stimuli Responsive Hydrogels

**Figure 3:**
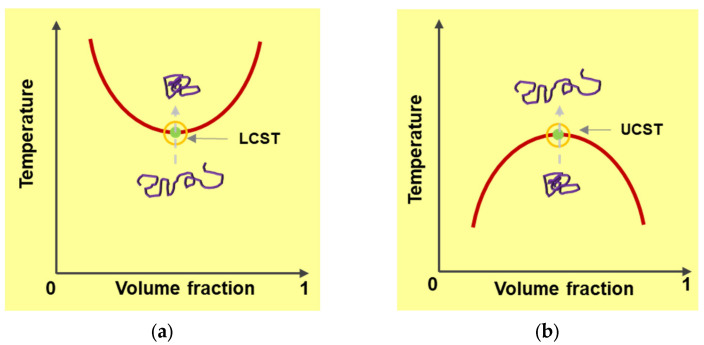
A polymer solution with a LCST starts to phase separate when temperature rises to its LCST (**a**). A polymer with UCST goes through the transition from insoluble to soluble state with increasing temperature approaching the critical point (**b**). Conformational transformations which occur during these transitions are also illustrated by darker color lines.

Materials that switch their molecular configuration in response to external temperature, pH, ionic strength, or electromagnetic field changes are called “responsive materials”. Thermo-responsive polymers undergo phase transition when their solution temperature changes. Some polymers have a critical transition temperature at which immediate switching of polymer solubility occurs. There are two manifestations of this critical temperature, lower critical solution temperature (LCST), and upper critical solution temperature (UCST). A polymer solution with an LCST starts to phase separate (dissolving of the polymer) when temperature rises to its LCST. A polymer with UCST goes through the transition from insoluble to soluble state with increasing temperature approaching the critical point. Such phase transition behavior of polymer chains across the phase separation boundary causes swelling and deswelling to occur (Figure 3). 

If an aqueously soluble complex (nonlinear) polymer absorbs a large amount of water, a hydrogel is formed. This type of polymer does not dissolve, but swells in the solvent, because the network in hydrogel provides a resisting force against its polymer chains to be completely separated by water molecules. When dissolving of polymer chains and stretching of cross-links equilibrate, hydrogel structure and the amount of water taken become stable.

**Figure 4:**
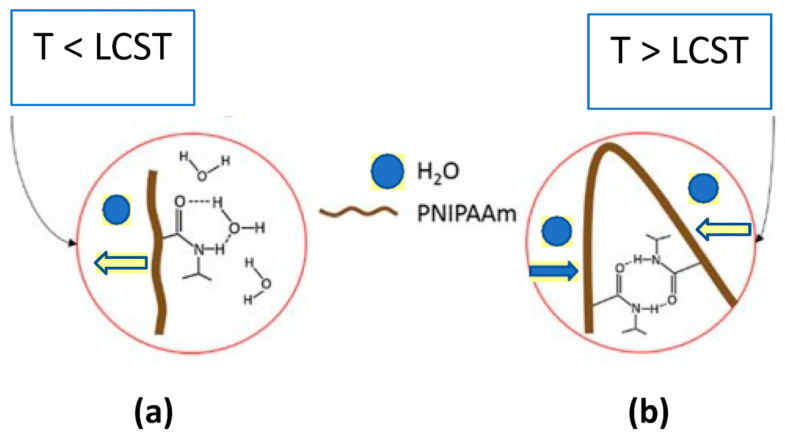
For temperature-responsive hydrogel with LCST (**a**), phase transition takes place between the polymeric component of the gel and the solvent absorbed when temperature exceeds the LCST (T > LCST). Such polymer cross-linking becomes insoluble, leading to the deswelling of hydrogel (**b**). Such swelling/deswelling (conformational) rearrangements within the polymer molecules are depicted by block arrows.

For temperature-responsive hydrogel with LCST, a phase transition takes place between the polymeric component of the gel and the solvent absorbed when temperature exceeds the LCST (T > LCST). Such polymer cross-linking becomes insoluble, leading to the deswelling of the hydrogel (Figure 4).

**Figure 5:**
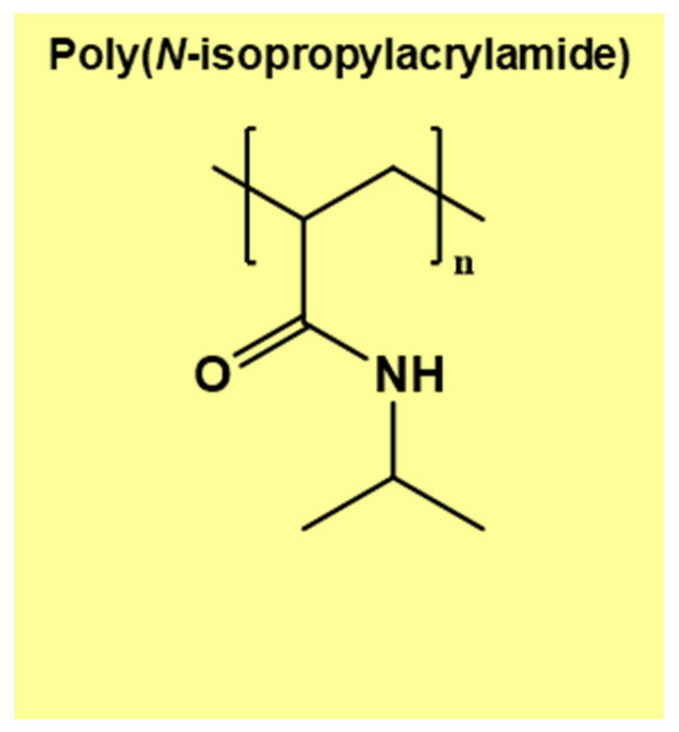
The chemical structure of PNIPAM.

Thermo-responsive hydrogel poly(N-isopropylacrylamide) (PNIPAM) has a 32 °C LCST, which is very close to human physiological temperature, and as such, offers potential for biological and medical applications. The chemical structure of PNIPAM is shown in Figure 5. Hydrogen bonding between the NH groups (Figure 5) and any water molecules introduced transform the linear molecules into coil conformation below the LCST (Figure 4) [54,55,56]. At temperatures higher than the LCST, the conformation of the polymer chains transforms into folded globules owing to a strong hydrophobic effect caused by carbon backbone and isopropyl groups [54,55]. In PNIPAM aqueous solution, hydrophobicity of PNIPAM increases, leading to phase separation. As explained earlier, hydrogen bonding between the amide groups and water is weakened when higher molecular kinetic energy rising from increased temperature exceeds that for hydrogen bonding. 

## 5. Sample Experiments and Results

**Figure 6:**
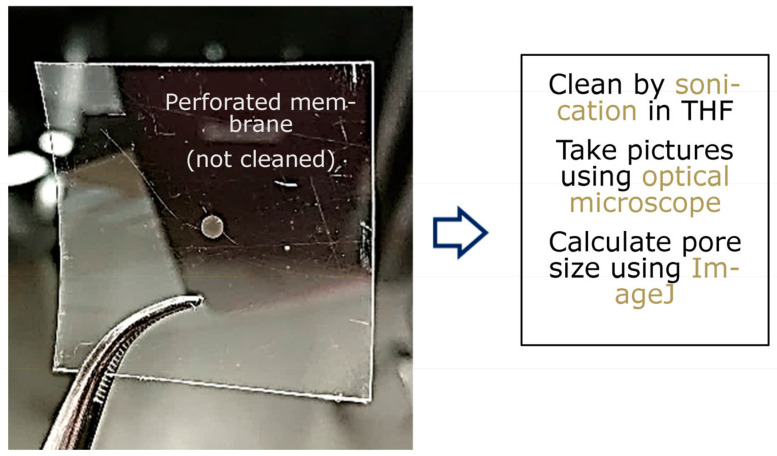
Tetrahydrofuran (THF) is used to clean the ablated polymer films. The pore morphology can be analyzed by optical microscopy, and the average pore sizes can be calculated by using image analysis software, such as ImageJ and Excel.

An LPX 240i excimer laser (Lambda Physik) is employed with Kr and F_2_ gases to generate 248 nm wavelength UV light by using the high constant voltage mode (at 26 kV). Here, d = 50, 75, 100, and 125 μm thick poly(ethylene terephthalate) (PET) (Melinex-S^®^ DuPont, Wilmington, Delaware, USA) films are used as the carrier films. Mesh (m) = 325 (pore size 43 µm), mesh 400 (pore size 31 µm), and mesh 500 (pore size 25 µm) grids are used as template masks. Tetrahydrofuran (THF) is used to clean the ablated polymer films. The pore morphology can be analyzed by optical microscopy, and the average pore sizes can be calculated by using image analysis software, such as ImageJ [57] and Excel (Figure 6).

**Figure 7:**
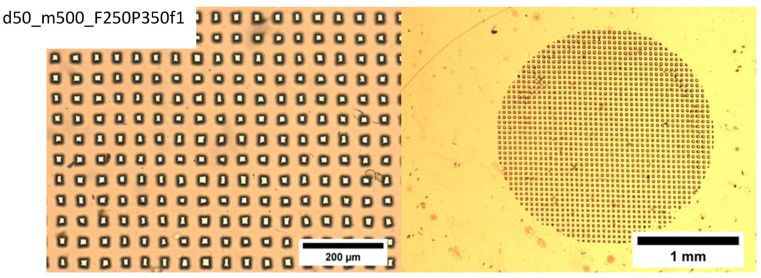

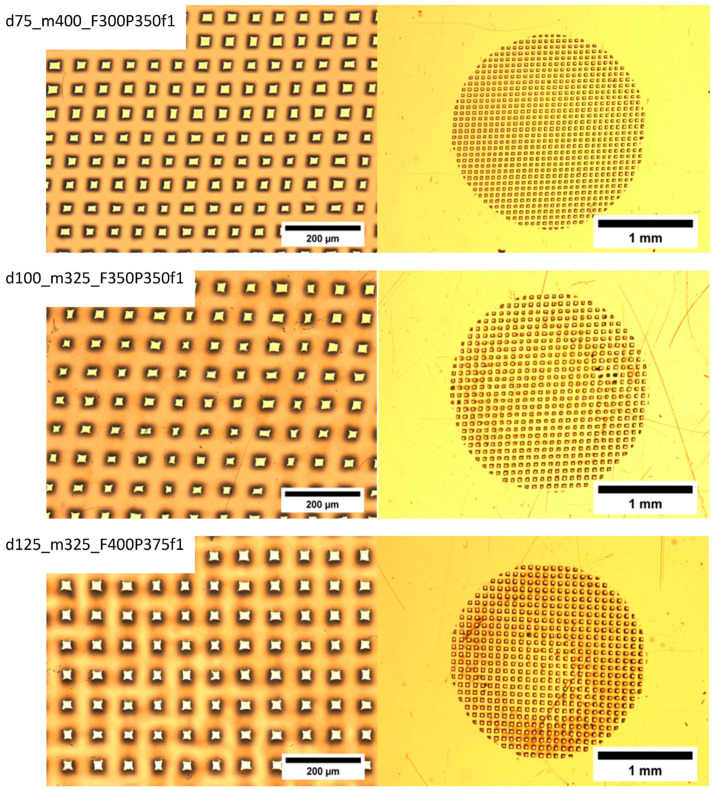
Typical ablated films obtained with different operation parameters (P) number of pulses, (F) fluence (energy/area) (mJ/cm^2^), (f) laser frequency (Hz), film thicknesses (d), and mesh sizes (m). Ablation threshold for PET ablated at 248 nm UV is 30 mJ/cm^2^. The black areas surrounding the pores are laser heat-affected areas.

The following laser operation parameters are monitored during film ablation: F, fluence (energy/area) (mJ/cm^2^) (ablation threshold for PET ablated at 248 nm UV is 30 mJ/cm^2^), P, number of pulses, and f, laser frequency (Hz). Typical ablated films are shown in Figure 7 for different operation parameters (F, f), film thicknesses (d) and mesh sizes (m). The black areas surrounding the pores are laser heat-affected areas.

Examination of Figure 7 reveals that increasing the mesh opening size (decreasing the mesh number, m) results in larger average pore size, cleaner edges, more uniform shape, well-defined pores, and a smaller affected area (dark area).

**Figure 8:**
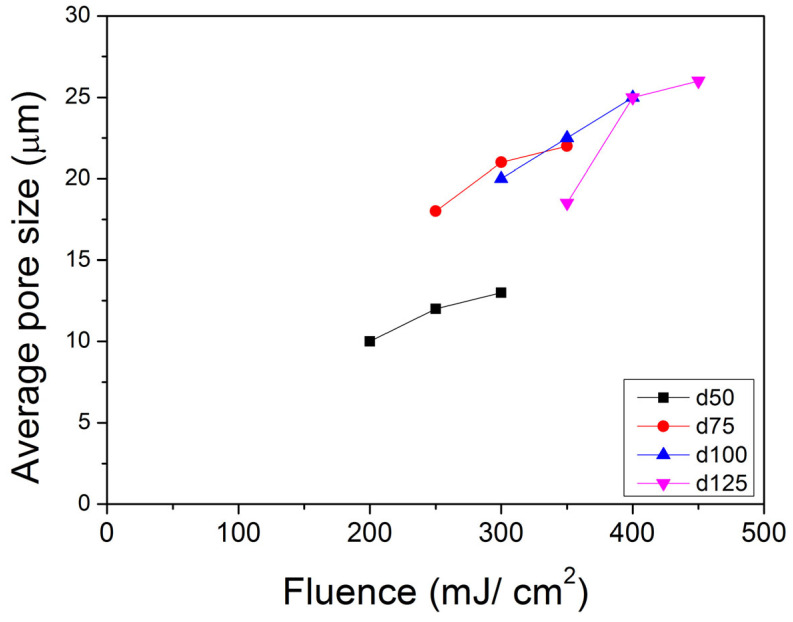
Average pore size (APS) vs. fluence (F) behavior of support membranes of four different thicknesses (d): 50 μm, 75 μm, 100 μm, and 125 μm. Each membrane thickness is studied under three F levels by using four pores for each condition. The APS standard deviations were less than 3 μm and typically 1.5–2.0 μm.

Work by Wu and Sancaktar [26] reported on the behavior of the average pore size (APS) vs. the number of pulses (P) of support membranes of four different thicknesses (d): 50 μm, 75 μm, 100 μm, and 125 μm. Each membrane thickness is studied under three fluence (F) levels by using four pores for each condition. Their results show that “pore open” P drops when F increases. Once the pores open, APS will remain almost the same. The final average pore size increases with increasing F, and more energy of the laser beam is needed to open pores for thicker films (Figure 8). 

**Figure 9:**
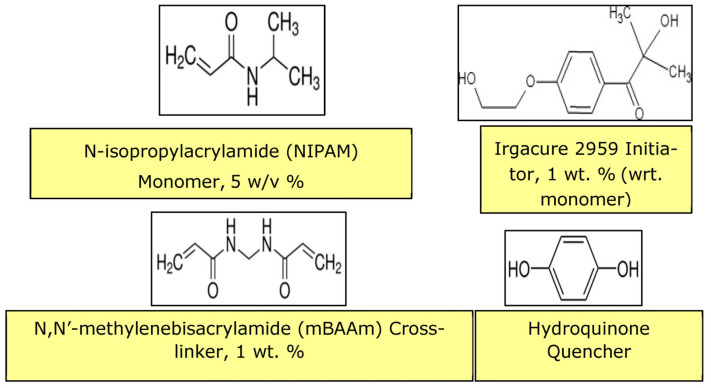
Material components for hydrogel PLP of PNIPAM.

**Figure 10:**
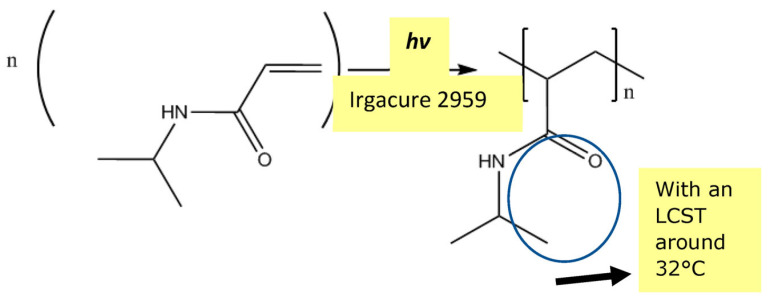
Pulsed laser polymerization (PLP) of PNIPAM.

**Figure 11:**
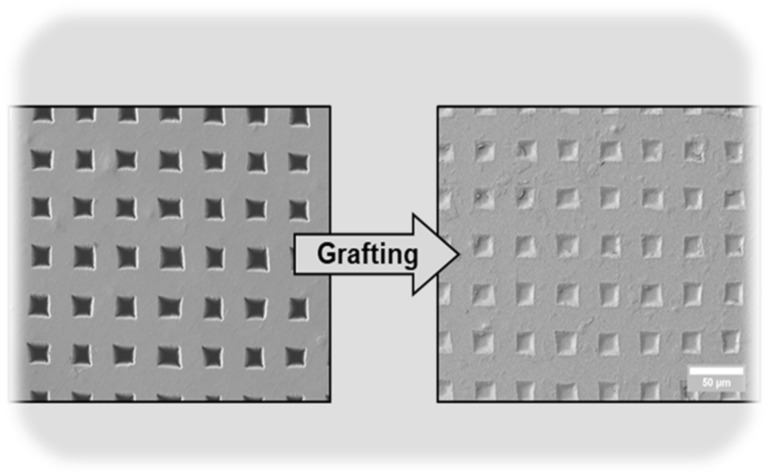
A PET membrane fully grafted by PNIPAM pulsed laser polymerization.

The pores created by laser ablation can be grafted with PNIPAM hydrogel by using the same laser to perform a bottom-up grafting-from type PLP (Figure 9, Figure 10 and Figure 11) at 5 mJ/cm^2^ in order to achieve the desired transport function controlled by temperature. O_2_ is removed from the grafting solution by bubbling N_2_ and from the grafting chamber by purging with N_2_. 

Wu and Sancaktar [26] used a set of laser frequencies and pulse numbers (Table 1, Figure 11) to obtain the desired hydrogel grafting density and the extent of cross-linking, which ultimately provide controlled transport by smart gating. The cross-linker concentration typically has a stronger effect on the temperature responsiveness. 

**Figure 12:**
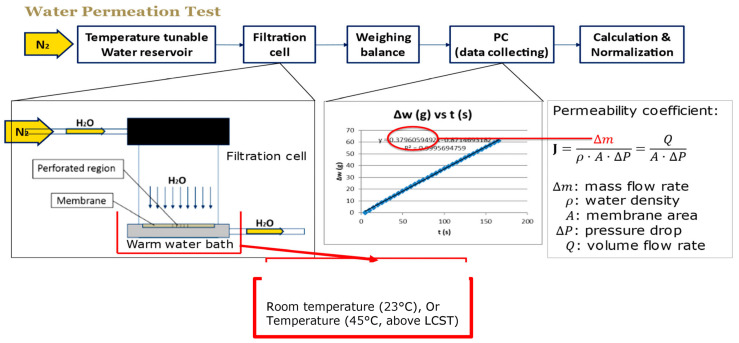
Measurement of water permeability for membranes manufactured by using different grafting conditions.

Wu and Sancaktar [26] determined the water permeability of membranes manufactured by using different grafting conditions with a filtration cell (Amicon 8010, Millipore, Burlington, MA, USA) which was kept in a temperature–controlled water bath. A preheated water reservoir upstream of the cell was pressurized by a nitrogen tank between 0.034 MPa (5 psi) and 0.31 MPa (45 psi) depending on the degree of grafting. A balance weighed the outcoming water in order to calculate water permeability coefficient from the slope of the weight-time curve at 23 °C and 45 °C by using the equation

(4) $$\;J=\frac{m}{\rho \cdot A\cdot \Delta P}\;,$$

where *m* is the slope of weight versus time curve, *ρ* is the density of water (1 mg/mL), *A* is the area of perforated region, and Δ*P* is the applied pressure. This procedure is summarized in Figure 12.

These experiments also illustrate high mechanical integrity and strong attachment of the grafted hydrogel inside the pores of the membranes, which sustain pressures up to 0.31 MPa. We note that previous works by Sancaktar et al. have shown increased adhesion to substrate (in this case, pore) surfaces after excimer laser irradiation, mostly due to the creation of advantageous surface topography (mechanical adhesion) and increased surface polarity [58,59,60,61,62].

Hagen–Poiseuille’s equation can be used to calculate the average pore size for grafted membranes,

(5) $$\Phi =2r=\sqrt[{4}]{\frac{8\mu Q}{\pi N_{p}}\cdot \frac{\Delta x}{\Delta P}}\;,$$

where ***Φ*** is the equivalent average pore diameter, ***r*** is the average pore radius, μ is water viscosity, ***Q*** is the volume flow rate through membrane, Δx is the membrane thickness, ***N_p_*** is the number of pores, and ΔP is the applied pressure. The permeability coefficient should be normalized w.r.t. viscosity (***μ***) due to the possible variations in flux viscosity resulting from temperature variations, as well as w.r.t. pore density (***ρ***′) and pore size (***Φ***) due to possible variations in those parameters, to result in the normalized permeability coefficient

(6) $$J\prime \prime =\frac{J}{\rho \prime \cdot \Phi^{4}}\propto \frac{1}{\mu }\cdot \frac{1}{d}\;$$

**Figure 13:**
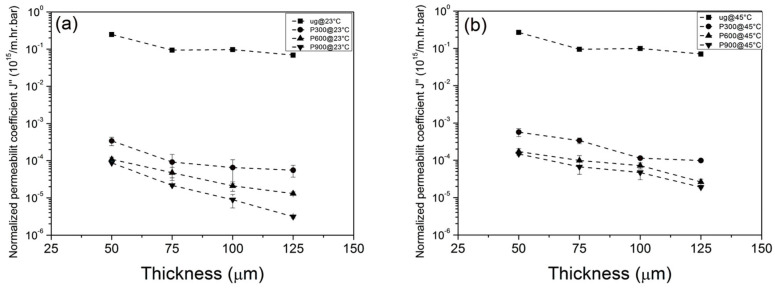
The relation between normalized permeability coefficient and membrane thickness for PNIPAM-grafted PET carrier membranes with different thicknesses. (**a**) results at 23 °C; (**b**) results at 45 °C.

Equation (5) provides the relation between normalized permeability coefficient and membrane thickness, which is illustrated in Figure 13 for PNIPAM-grafted PET carrier membranes with different thicknesses.

Examination of Figure 13 reveals that the permeability coefficient decreases with increasing membrane thickness and number of pulses. Comparison of Figure 13a (results at 23 °C) with Figure 13b (results at 45 °C) reveals up to ~fivefold increase in permeability coefficient for water flux when the temperature goes above the LCST (~32 °C). We note that the work by Wu and Sancaktar also revealed that the film thickness has little or no effect on PLP kinetics with similar cross-link structure obtained with PNIPEM grafted in different thickness support membranes.

## 6. Conclusions

It has been shown that 248 nm KrF excimer laser can be used to develop stimuli-responsive polymer-based membranes for controlled transport applications. For this purpose, well-defined/shaped and orderly pores are created on commercially available polymer films by ablation by using an excimer laser. The same laser is used subsequently for energetic grafting and polymerization of a responsive hydrogel polymer inside the pores. This is a fast process which offers flexible tuning of (water) flux by using laser operation parameters, and the smart membranes allow controllable solute transport. Creation of membranes with 600 nm to 25 μm pore sizes by using the laser through different metal mesh templates with different laser pulses and fluences has been demonstrated. These parameters need to be optimized to obtain the desired pore size. Pore size increases with increasing fluence and the number of pulses, as well as the mesh size. 

The pores created by laser ablation can be grafted with an appropriate hydrogel using the same laser to perform a bottom-up grafting-from type PLP in order to achieve the desired transport function controlled by a stimulus. For this purpose, a set of laser frequencies and pulse numbers needs to be determined to obtain the desired hydrogel grafting density and the extent of cross-linking, which ultimately provide controlled transport by smart gating. The PLP process is extremely fast (few seconds) and provides higher water permeability above the lower critical solution temperature (LCST) of the hydrogel.

The monomer and cross-linker concentrations in the grafting solution need to be optimized in order to control the network growth inside the support membrane pores. The cross-linker concentration typically has stronger effect on the temperature responsiveness. In addition to temperature responsiveness, the PLP process can be extended to different unsaturated monomers, which can be polymerized by the free radical process. For example, poly(acrylic acid) can be the grafted to provide pH responsiveness to membranes. 

As for the effects of thickness, a decreasing trend is observed in the permeability coefficient with increasing thickness. Comparison of results at 23 °C with results at 45 °C reveals up to ~fivefold increase in permeability coefficient for water flux when the temperature goes above the LCST (~32 °C). Furthermore, the film thickness has little or no effect on PLP kinetics. 

The experimental results have shown that membranes fabricated by using an excimer laser are excellent choices for applications where flow uniformity is the prime requirement, as they possess uniform pore sizes and distribution.

## Figures and Tables

**Table 1:** Operation times for various grafting conditions of PNIPAM pulsed laser polymerization.

Grafting Condition	Operation Time (s)
F5P300f25	12
F5P600f25	24
F5P900f25	36
